# The use of an online Epworth Sleepiness Scale to assess excessive daytime sleepiness

**DOI:** 10.1007/s11325-016-1417-x

**Published:** 2016-11-12

**Authors:** Jennifer Boyes, Panagis Drakatos, Ian Jarrold, Judy Smith, Joerg Steier

**Affiliations:** 1 Faculty of Life Sciences and Medicine, King’s College London, London, UK; 2 Lane Fox Respiratory Unit/Sleep Disorders Centre, Guy’s and St Thomas’ NHS Foundation Trust, Ground Floor, South Wing, Westminster Bridge Road, London, SE1 7EH UK; 3 British Lung Foundation, London,, UK

**Keywords:** Sleep apnea, Sleep disorder, Questionnaire, Upper airway

## Abstract

**Purpose:**

Excessive daytime sleepiness is the most common complaint reported in sleep clinics. We hypothesised that utilising modern media to deliver an online Epworth Sleepiness Scale, age- and gender-related differences in subjective daytime sleepiness could be assessed.

**Methods:**

Age, gender and online Epworth Sleepiness Scale (range 0–24 points) of 39,448 subjects were recorded between January 2013 and November 2015.

**Results:**

A significant trend, for males but not females, was found between age and Epworth score (*p* < 0.001). Average scores were higher for female subjects in their 1st and 2nd (*p* = 0.014), 3rd (*p* < 0.011) and 4th lifetime decade (*p* = 0.011), whereas male subjects conveyed significantly higher levels of sleepiness in their 7th lifetime decade (*p* < 0.001). Individual item analysis found differences between gender; females scored significantly higher than males in items 1, 4 and 5, while male subjects had higher scores for items 3, 6, 7 and 8. Lowest levels of sleepiness were reported for item 8 and highest scores for item 5.

**Conclusions:**

The use of an online Epworth Sleepiness Scale identifies gender- and age-specific differences and facilitates new pathways in the delivery of chronic care.

## Introduction

Excessive daytime sleepiness (EDS) is a debilitating and potentially dangerous symptom that compromises cognitive performance, leads to poor productivity and can result in harm to the individual or to the general public [1]. EDS has been defined as an inability to stay awake and alert during the major waking periods of the day causing unintentional lapses into drowsiness or sleep occurring on a daily basis for more than 3 months [2]; it can affect up to 18 % of the general population [3].

Frequently, a cause of EDS cannot be identified, but there are many contributing factors, most commonly those which disturb sleep quality or quantity. Other risk factors for EDS include age, depression, shift work, metabolic and hormonal conditions and obesity [3]. EDS is a cardinal symptom for hypersomnia of central origin; it is highly prevalent in sleep disorders and multiple other health conditions [3]. Clinically, EDS is the most common complaint reported in sleep clinics [4] and is often associated with sleep apnea and periodic limb movement disorder (PLMD) [3].

Sleepiness reduces quality of life and can affect other people in situations where reactivity and alertness are required, such as for driving. EDS has a negative impact, economically and socially, on physical and mental health and safety and poses a substantial cost burden on the healthcare system. Clinicians need to be aware of the dangers of excessive sleepiness, discuss appropriate sleep hygiene and treat underlying risk factors [5].

It is difficult to quantify chronic symptoms like sleepiness; it requires the patient to report the quality of the symptom and its intensity. However, there are multiple methods that try to measure physiological aspects associated with sleepiness: polysomnography and the multiple sleep latency test (MSLT) are frequently combined to measure the mean sleep latency during standardised naps, as an objective marker for sleepiness. On the other hand, the Epworth Sleepiness Scale (ESS) is a validated questionnaire to assess subjective sleepiness and sleep propensity [6, 7] with important clinical utilisation [8]. A pictorial version of the ESS has been developed in the past to overcome language barriers and poor literacy [9].

To assess the outreach and resonance of a questionnaire validated for subjective daytime sleepiness on a large scale, we utilised an online version of the ESS and these data were used to compare age- and gender-related differences in EDS.

## Patients and methods

This study was a collaborative project between the British Lung Foundation (BLF), King’s College London and King’s Health Partners at Guy’s and St Thomas’ NHS Hospital Foundation Trust, London, UK. Interested individuals accessed the BLF webpage with a link to an English version of the ESS, as part of the BLF campaign to raise awareness of obstructive sleep apnea (OSA). A brief explanation to the eight domains of the ESS was provided. Gender (male/female) and age (lifetime decade) were recorded, followed by an explanation of the ESS scores. Concurrent with other ESS studies, a score >10 points was used to define EDS [9].

### Data acquisition

Data acquisition began in January 2013 and finished in November 2015. A total of 39,454 people participated in the study. However, due to incomplete data transfer, 6 subjects were excluded from the statistical analysis, making a study size of 39,448 subjects. Final scores and individual item scores were recorded and compared to previous data using the standard ESS and the pictorial ESS [6, 9, 10]. The impact of age and gender on total and individual items’ scores was further explored. Based on the indicated gender and age group, subjects were categorised into male, female and lifetime decades, with the exception of age 0–19 and >80 years. These age groups were combined into single age categories, respectively.

### Epworth Sleepiness Scale

The ESS is a short, self-administered questionnaire that consists of eight questions asking to rate how likely it is to fall asleep in everyday situations (each question can be scored from 0 to 3 points; ‘0’ indicates no sleepiness, ‘3’ indicates significant sleepiness). It provides a total score which has been shown to relate to the subject’s level of daytime sleepiness (total score range 0–24 points). Originally, it was validated in patients with OSA; however, it is now commonly used for patients suffering from sleep-related problems [9].

### Statistical analysis

Statistical analysis of the data was performed using SPSS (Version 22.0.0, IBM, New York/NY, USA). Data were reported as mean (standard deviation, SD), unless otherwise indicated. To ensure that the data were valid, the following steps were taken:

1. The BLF website had an introduction explaining the purpose of the online questionnaire and used character checks when collecting the data, such as gender and age, i.e. non-sense variables could not be selected.
2. Format checks were in place to ensure that data were consistent, e.g. DOB DD/MM/YYYY. Also, a presence check meant all fields had to be complete in order to submit the questionnaire.
3. To ensure data were not duplicated, i.e. the same person did not answer the questionnaire twice, the data underwent a uniqueness check to ensure each data set was unique and no two submissions had the same name and DOB.
4. An automated check of IP addresses was run to detect submission of multiple questionnaires from the same computer.
5. Filter checks looked for identical records.
6. After the data had been collected, an MS Excel sheet (Microsoft, Seattle/WA, USA) was generated and we examined this to ensure all data sets were complete. We implemented consistency and range checks on the categorical and discrete variables, respectively, in the Excel sheet, to confirm that data were within the correct parameters.
7. We also conducted batch total checks as we analysed the data to ensure we did not miss a dataset in our analysis.

As there was no financial or material incentive for answering the questionnaire, there was a low risk of someone submitting multiple questionnaires. Subsequent to testing for normality, means were compared using the Student’s *t* test; the Mann Whitney *U* test was used to compare non-normally distributed data. Comparisons between age group and ESS scores of different items were undertaken using one-way ANOVA or the Kruskal-Wallis test, if there was a non-Gaussian distribution. The *p* value for the trend between sleepiness and age was calculated using simple and multiple linear regression analysis to further describe age and gender interaction. To assess the internal consistency of the questionnaire, Cronbach’s statistic alpha was used for item analysis. A level of significance was considered with a *p* < 0.05.

## Results

Data were collected from 39,448 participants; 55 % of the subjects were male and 45 % were female. More than half of the responses (51.5 %) scored >10 points on the ESS. There was a significant trend for higher age to be associated with higher ESS scores for male subjects (*p* < 0.001), but not for female participants (*p* = 0.312). Gender distribution varied between the age categories; there were more female responders up to the age of 30 years, while there were more male subjects in the older age categories (Table 1).

**Table 1:** Age groups, gender distribution and ESS

Age groups	0–19	20–29	30–39	40–49	50–59	60–69	70–79	≥80	Total
M F (% of M)	317 435 (42.2)	1961 2073 (48.6)	3459 2702 (56.1)	5049 4288 (54.1)	5541 4735 (53.9)	3691 2573 (58.9)	1484 703 (67.9)	298 139 (68.2)	21,800 17,648 (55.3)
Total (% of all)	752 (1.9)	4034 (10.2)	6161 (15.6)	9337 (23.7)	10,276 (26.0)	6264 (15.9)	2187 (5.5)	437 (1.1)	39,448 (100)
ESS (mean, SD)	10.1 (5.9)	10.1 (5.3)	10.6 (5.3)	11.1 (5.4)	11.0 (5.5)	10.6 (5.5)	10.5 (5.4)	11.1 (5.7)	10.8 (5.0)

Age groups are presented in years

*M* male, *F* female

**Fig. 1:**
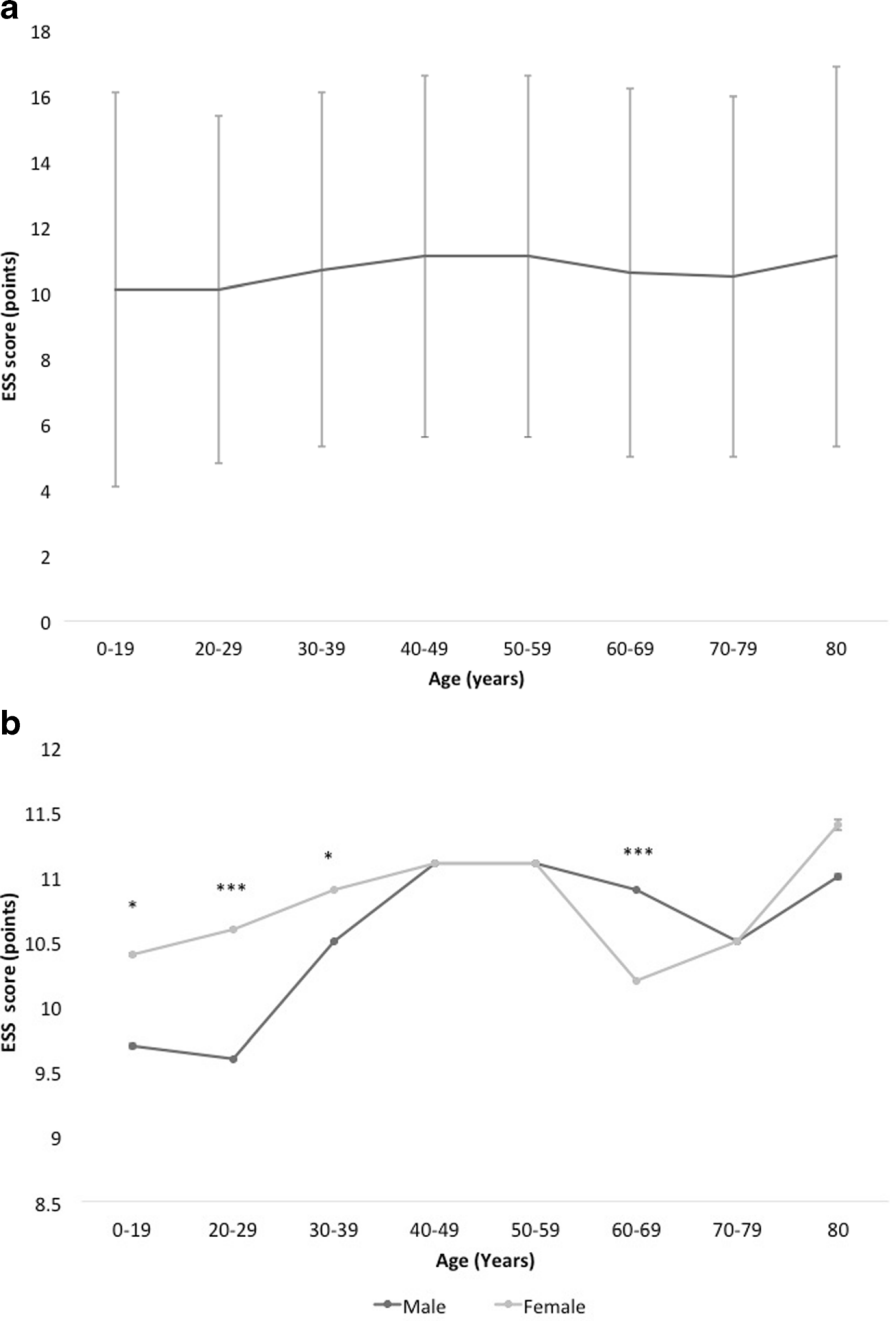
**a** Epworth Sleepiness Scale (ESS) vs age, total group (*n* = 39,448). Data are presented as mean (SD), *p* < 0.001. **b** Epworth Sleepiness Scale (ESS) vs age, grouped by gender. Data are presented as mean (SE). Male (*n* = 21,800), *black line*; female (*n* = 17,648), *grey line*. *SE* standard error (small error bars). *p < 0.05, ***p < 0.001

Female subjects tended to score significantly higher compared to male subjects up to their 4th lifetime decade, while male subjects scored significantly higher in their 7th lifetime decade (Fig. 1a, b).

### Item analysis

**Fig. 2:**
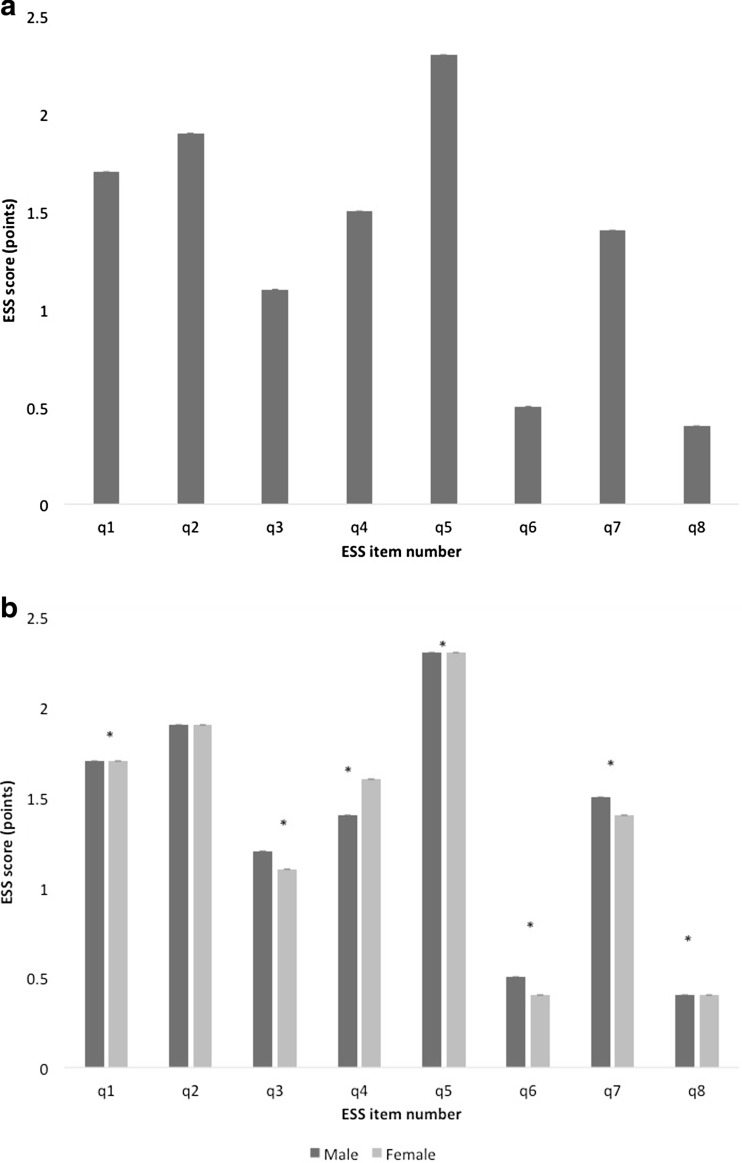
**a** ESS, mean score for all eight items. Data are shown as mean (SE), *n* = 39,448. *ESS* Epworth Sleepiness Scale; *SE* standard error (error bars small). **b** ESS item scores according to gender. Data are shown as mean (SE). Male subjects (*n* = 21,800), *dark grey bars*; female subjects (*n* = 17,648), *light grey bars*. *p < 0.05. *ESS* Epworth Sleepiness Scale; *SE* standard error (*small error bars*)

An item analysis of the individual responses to each question of the ESS was conducted. Item 5, ‘Lying down to rest in the afternoon when circumstances permit’, scored highest, while item 8, ‘in a car, while stopped for a few minutes in traffic’, scored lowest for both genders (*p* < 0.001) (Fig. 2a, b).

Male subjects scored significantly higher in half of the items (items 3, 6, 7, 8), while female subjects scored higher in items 1, 4 and 5. A rank sum analysis of each item was conducted and compared to previous work [6, 9, 10]; there was good agreement with previous data (Table 2).

**Table 2:** Rank sum analysis of items of the ESS and pictorial ESS, from high to low scores

Rank sum	Current study, ESS	Drakatos et al., pictorial ESS [8]	Ghiassi et al., pictorial ESS [9]	Ghiassi et al., ESS [9]	Johns, ESS [6]
1	Q5 2.29 (0.96)	Q5 2.18 (0.93)	Q5 2.14 (0.95)	Q5 2.12 (0.95)	Q5 2.47 (0.8)
2	Q2 1.90 (1.00)	Q2 1.72 (1.00)	Q2 1.50 (0.92)	Q2 1.50 (0.89)	Q4 1.53 (1.12)
3	Q1 1.69 (1.05)	Q1 1.37 (1.00)	Q1 1.24 (0.93)	Q7 1.28 (0.97)	Q1 1.48 (1.05)
4	Q4 1.52 (1.14)	Q4 1.29 (1.07)	Q7 1.22 (1.00)	Q1 1.18 (0.88)	Q7 1.31 (1.05)
5	Q7 1.42 (1.08)	Q7 1.16 (1.03)	Q4 1.08 (1.02)	Q4 1.03 (1.01)	Q2 1.30 (1.11)
6	Q3 1.14 (1.05)	Q3 1.02 (0.95)	Q3 1.02 (1.13)	Q3 0.89 (0.80)	Q3 1.30 (1.02)
7	Q6 0.45 (0.77)	Q6 0.34 (0.68)	Q6 0.30 (0.62)	Q6 0.27 (0.49)	Q8 0.47 (0.87)
8	Q8 0.38 (0.76)	Q8 0.27 (0.64)	Q8 0.28 (0.65)	Q8 0.24 (0.53)	Q6 0.39 (0.76)
Total ESS	10.79 (5.49)	9.35 (7.3)	8.78 (7.22)	8.51 (6.52)	10.25 (7.78)

Data are presented as mean (SD)

*SD* standard deviation

Internal consistency of the items in the questionnaire was measured using the Cronbach’s statistic alpha, and item scores from all subjects were included. The Cronbach’s alpha was 0.85 for the total of 39.448 participants, and the alpha value did not increase after deleting any of the items from the questionnaire. These results are similar to those reported by Johns in 1992 and indicate a high level of consistency between the eight items of the online version of ESS [11].

## Discussion

The use of an online version of the Epworth Sleepiness Scale enabled an average of 1160 subjects per month, over a 34-month period, to access information about their symptoms and assess their health concerns. In a large cohort of subjects using an open access online Epworth Sleepiness Scale, female subjects were sleepier in early life, compared to male subjects, who scored higher in their 7th lifetime decade.

Gender-specific variations in age pose the question of whether specific and extrinsic factors may contribute to subjective symptom perception and psychological coping [12–14]. The effect of age on symptom presentation has been controversially discussed. While a specific effect has previously been described by us using the pictorial ESS [9], and by others, specifically for male adults [15, 16], other studies have not found an association between age and EDS [14, 17, 18].

Hypothetically, gender-specific differences in EDS may be affected by hormonal influences and it has been shown that women with severe pre-menstrual syndrome report insomnia and increased fatigue during the luteal phase; subtle changes to sleep structure are linked to fluctuations in progesterone. As the age category in which females have statistically higher ESS scores incorporates the typical ages a woman would start a family, it seems likely that these factors might contribute [12].

Although differences were observed between genders in respect to the percentage of sleepy subjects, there was no significant overall trend for females to be sleepier with higher age; however, older male subjects scored higher and similar studies have found that male subjects scored significantly higher overall. A potential explanation is offered by some studies claiming that women have a comparatively higher basic wake drive and hence experience less sleepiness and score lower on the ESS [19]. Also, male subjects are more prone to being overweight and having common sleep disorders, like obstructive sleep apnoea [13, 20]. Furthermore, objective data report significantly shorter sleep latencies in men [21]. When these points are interpreted within the context described in the ESS, it is to be expected that subjects feel more drowsy during natural physiological soporific conditions, e.g. when lying down to rest in the afternoon, compared to the alerting situation of being a passenger in a car stopped in traffic [6, 9, 10]. With regards to gender-related differences in item scores, a scientific explanation would require a more comprehensive approach including differences in brain physiology, circadian rhythm and socioeconomic and demographic factors.

The highest level of sleepiness from our data were reported in the elderly (>80 years) which might be due to a selection bias. However, wakefulness and vigilance in the elderly are affected by reduced activity levels, daytime napping, medical conditions, bereavement, degenerative changes in the central nervous system and other medical conditions [9, 20, 22]; in addition, changes in the circadian rhythm may occur in ageing people. A higher prevalence of sleep disorders such as OSA, insomnia, PLMD and other co-morbidities linked with organic problems in the elderly may further impact on the presentation of EDS [3, 9, 20].

Variation was observed between the rank sum of the individual questions in the ESS when compared to other studies. Item 5 of the ESS, which addresses how likely people are to fall asleep when lying down in the afternoon, ranked first in the current and all other studies [6, 9, 10]. This is likely to be a result of the soporific nature of this situation in which it is accepted that even normal people may fall asleep [6]. In all but one study [6], item 2 ‘sitting watching TV’ ranked second. This variation may be explained by the increase in access and ownership of TVs since the 1990s, accounting for its higher rank in the more recent studies which were all conducted post-2010 [23], as well as in a different cohort studied in by Johns [6]. Item 1 ‘sitting and reading’, item 4 ‘as a passenger in a car’ and item 7 ‘sitting quietly after lunch without alcohol’ were between the 3rd and 5th rank. These items were expected to rank reasonably high due to the lack of situational stimulation which accounts for their sleep-inducing nature [6]. The variation in the rank sum position of these items is likely to be due to differences in lifestyles and group selection of the different studies. However, there was concordance across all studies for item 3 ‘sitting inactive in a public place e.g. a theatre’ as the 6th rank sum. All studies, except Johns [6], had item 6 ‘sitting and talking to someone’ and item 8 ‘in a car while stopped for a few minutes in traffic’ as 7th and 8th rank sums, respectively; these two were swapped in Johns’ study [6, 9, 10]. These two items were expected to be ranked lower due to the presence of stimuli in the respective environment. Some of the differences in the rank sums between the studies may be attributed to patient selection. It is interesting to note that the online pictorial ESS and the online ESS had the same rank sum item order. This underlines the consistency of online data collection and the repeatability of such studies. It also shows that the pictorial and the original ESS result in similar scores, as described by Ghiassi et al. [10].

The use of the Internet in recent years and an increased smartphone ownership to over a quarter of the global population predicted to rise to more than a 3rd by 2018 [24], which enhances the outreach of clinical services to the general population. Modern media does not only promote access to healthcare information but also facilitates the collection of a large amount of data, including demographics, in a relatively short time. These data may be used to identify individuals at risk of undiagnosed sleep disorders emphasising the progressive role technology has in aiding doctors and research. The ESS on a public website accompanied by information concerning sleep disorders, specifically OSA, increases public awareness about factors associated with sleep disorders which may help to improve time to diagnosis and treatment. It is not unthinkable that such information, protected from unauthorised access, could be used to facilitate and speed up clinic visits in future or enable driving licencing agencies to screen for ‘fitness to drive’.

### Limitations to this study

Participants were self-selected resulting in a responder bias, and there was a greater response from male subjects. This may be due to a higher prevalence of sleep disorders in male subjects [13]; being more affected by EDS could make males more likely to research their symptoms on the internet. A greater proportion of responders was also found in the middle-aged and older age categories, potentially due to increased concerns about the effects of EDS, or potentially because of more available time to respond. More subjects under 30 years were female, and this may be due to women generally being more concerned about health. The lack of information on potential confounders such as shift work, body mass index (BMI), socioeconomic factors, witnessed apneas, ethnicity and mental health means that care should be taken when interpreting our data. The self-administered approach of the online ESS also allows data duplication, although several safety measures were in place to minimise this effect. A priori, a self-administered questionnaire is less likely to be completed compared to physician-administered, but this does not negate the value when used online, as initially intended [25]. As a result, the advantages for public health by using online media to expand the population base and support healthcare services in identifying sleep disorders and raising awareness about OSA outweigh these limitations. Based on the ease and volume of data gathered, and in addition to increasing smartphone ownership and progressive introduction of older adults to the internet social media, it has a potential role in similar future health-related research, with emphasis on demographic, social and clinical data to allow for a more comprehensive evaluation of the nature of EDS and to improve the delivery of healthcare services.

### Conclusion

The use of an online version of the ESS delivers fast, accurate and comparable results to the conventional ESS for a large audience. Although gender and age might influence sleepiness perception, the multi-factorial contributions to the symptom require further studies to confirm the observed differences conclusively.

## References

[1] McWhirter D, Bae C, Budur K (2007). The assessment, diagnosis, and treatment of excessive sleepiness: practical considerations for the psychiatrist. Psychiatry (Edgmont).

[2] Swanson LM, Arnedt J, Rosekind MR, Belenky G, Balkin TJ, Drake C (2011). Sleep disorders and work performance: findings from the 2008 National Sleep Foundation Sleep in America poll. J Sleep Res.

[3] Slater G, Steier J (2012). Excessive daytime sleepiness in sleep disorders. J Thorac Dis.

[4] Vgontzas AN, Kales A (1999). Sleep and its disorders. Annu Rev Med.

[5] Ratneswaran C, Kadhum M, Pengo MF, Steier J (2016). Sleep, obesity and physicians’ education. J Thorac Dis.

[6] Johns MW (1991). A new method for measuring daytime sleepiness: the Epworth sleepiness scale. Sleep.

[7] Fernandez-Mendoza J, Calhoun SL. (2015) Excessive daytime sleepiness: age, sleep, mood, and metabolic modulation. In book: Modulation of sleep by obesity, diabetes, age, and diet, edition: 1, Chapter: 21, Publisher: Elsevier Academic Press, Editors: Ronald R Watson, pp. 193–202

[8] Johns MW (1994). Sleepiness in different situations measured by the Epworth Sleepiness Scale. Sleep-New York.

[9] Drakatos P, Ghiassi R, Jarrold I, Harris J, Abidi A, Douiri A (2015). The use of an online pictorial Epworth Sleepiness Scale in the assessment of age and gender specific differences in excessive daytime sleepiness. J Thorac Dis.

[10] Ghiassi R, Murphy K, Cummin AR, Partridge MR (2011). Developing a pictorial Epworth Sleepiness Scale. Thorax.

[11] Johns MW (1992). Reliability and factor analysis of the Epworth Sleepiness Scale. Sleep.

[12] Lee KA, Baker FC, Newton KM, Ancoli-Israel S (2008). The influence of reproductive status and age on women’s sleep. J Women’s Health.

[13] Lin CM, Davidson TM, Ancoli-Israel S (2008). Gender differences in obstructive sleep apnea and treatment implications. Sleep Med Rev.

[14] Souza JC, Magna LA, Reimão R (2002). Excessive daytime sleepiness in Campo Grande general population, Brazil. Arq Neuropsiquiatr.

[15] Bixler E, Vgontzas A, Lin H, Calhoun S, Vela-Bueno A, Kales A (2005). Excessive daytime sleepiness in a general population sample: the role of sleep apnea, age, obesity, diabetes, and depression. J Clin Endocrinol Metab.

[16] Morrell MJ, Finn L, McMillan A (2012). The impact of ageing and sex on the association between sleepiness and sleep disordered breathing. Eur Respir J.

[17] Ohayon MM, Caulet M, Philip P, Guilleminault C, Priest RG (1997). How sleep and mental disorders are related to complaints of daytime sleepiness. Arch Intern Med.

[18] Roth T, Roehrs TA (1996). Etiologies and sequelae of excessive daytime sleepiness. Clin Ther.

[19] Kim H, Young T (2005). Subjective daytime sleepiness: dimensions and correlates in the general population. Sleep.

[20] Slater G, Pengo MF, Kosky C, Steier J (2013). Obesity as an independent predictor of subjective excessive daytime sleepiness. Respir Med.

[21] Punjabi NM, Bandeen-Roche K, Young T (2003). Predictors of objective sleep tendency in the general population. Sleep.

[22] Howard ME, Desai AV, Grunstein RR, Hukins C, Armstrong JG, Joffe D (2004). Sleepiness, sleep-disordered breathing, and accident risk factors in commercial vehicle drivers. Am J Respir Crit Care Med.

[23] BARB. (2016) Television ownership in private domestic households 1956–2014 (millions). http://www.barb.co.uk/resources/tv-ownership/. Accessed 2 Aug 2016

[24] eMarketer. (2014) 2 Billion consumers worldwide to get smart(phones) by 2016 http://www.emarketer.com/Article/2-Billion-Consumers-Worldwide-Smartphones-by-2016/1011694. Accessed 6 Jun 2016

[25] Damiani MF, Quaranta VN, Falcone VA, Gadaleta F, Maiellari M, Ranieri T (2013). The Epworth Sleepiness Scale: conventional self vs physician administration. Chest.

